# Supplementary material for: Demonstrating Tumor Vascular Disrupting Activity of the Small-Molecule Dihydronaphthalene Tubulin-Binding Agent OXi6196 as a Potential Therapeutic for Cancer Treatment
Source: Cancers (Basel). 2022 Aug 30;14(17):4208. doi: 10.3390/cancers14174208 (PMC9454770; doi:10.3390/cancers14174208)
Supplement: Supplementary file 1 [file cancers-14-04208-s001.zip › cancers-1836320-supplementary.pdf]

Supplementary Materials

Demonstrating Tumor Vascular Disrupting Activity of the Small-Molecule Dihydronaphthalene Tubulin-Binding Agent OXi6196 as a Potential Therapeutic for Cancer Treatment

Li Liu [1,2], Regan Schuetze [1], Jeni L. Gerberich [1], Ramona Lopez [1], Samuel O. Odutola [3], Rajendra P. Tanpure [3], Amanda K. Charlton-Sevcik [3], Justin K. Tidmore [3], Emily A.-S. Taylor [3], Payal Kapur [2,4], Hans Hammers [2,5], Mary Lynn Trawick [3,†], Kevin G. Pinney [3,†], and Ralph P. Mason [1,2,*]

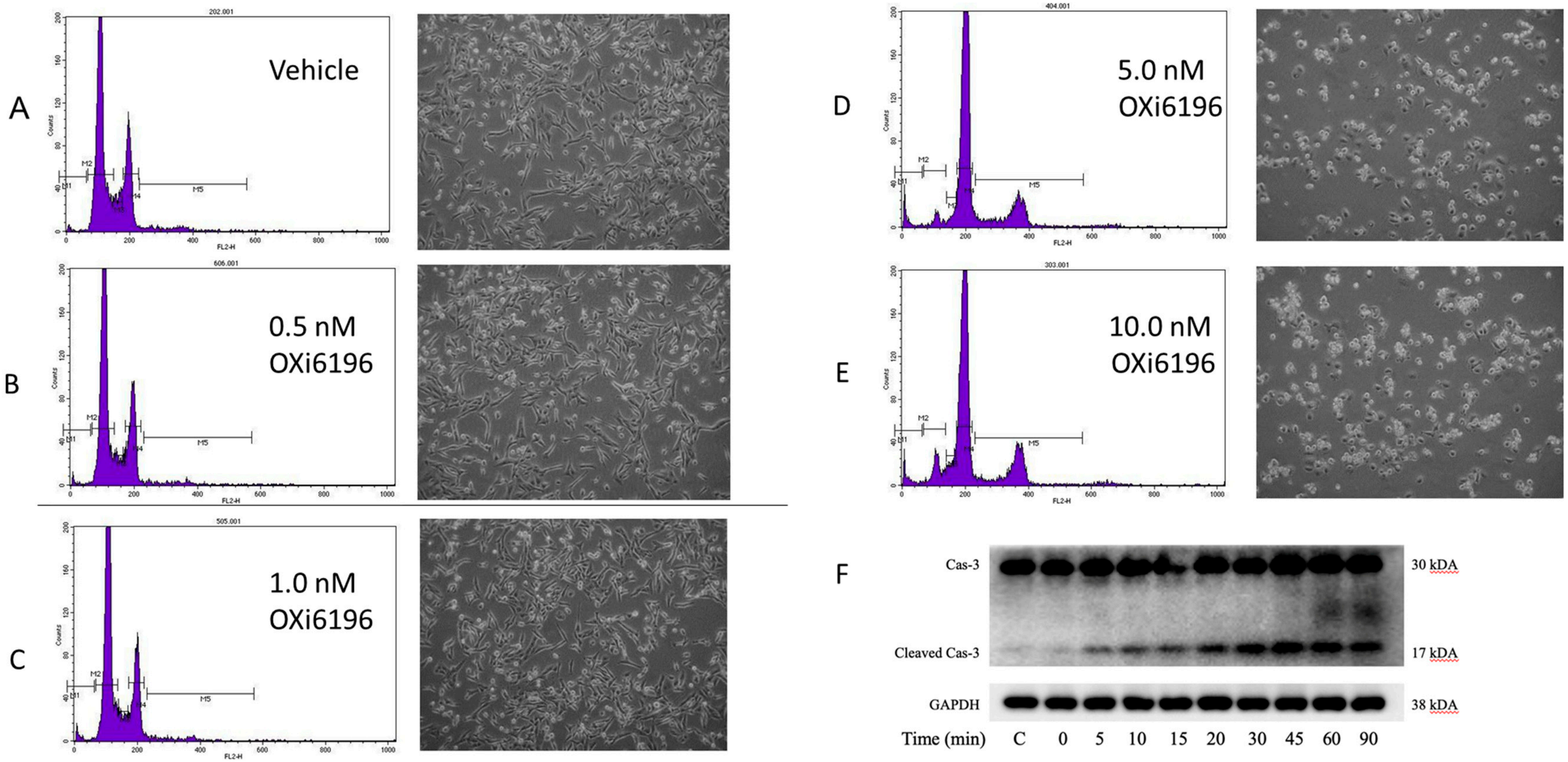

Figure S1: Flow cytometry of MDA-MB-231 cells treated with A) vehicle control, B) 0.5 nM, C) 1.0 nM, D) 5.0 nM, E) 10.0 nM OXi6196. For concentrations ≥ 5 nM G2/M blockade was observed and cells were round and lifting from substrate. F) Time-dependent cleavage of caspase-3 in MDA-MB-231 cells with 10 nM OXi6196 treatment.

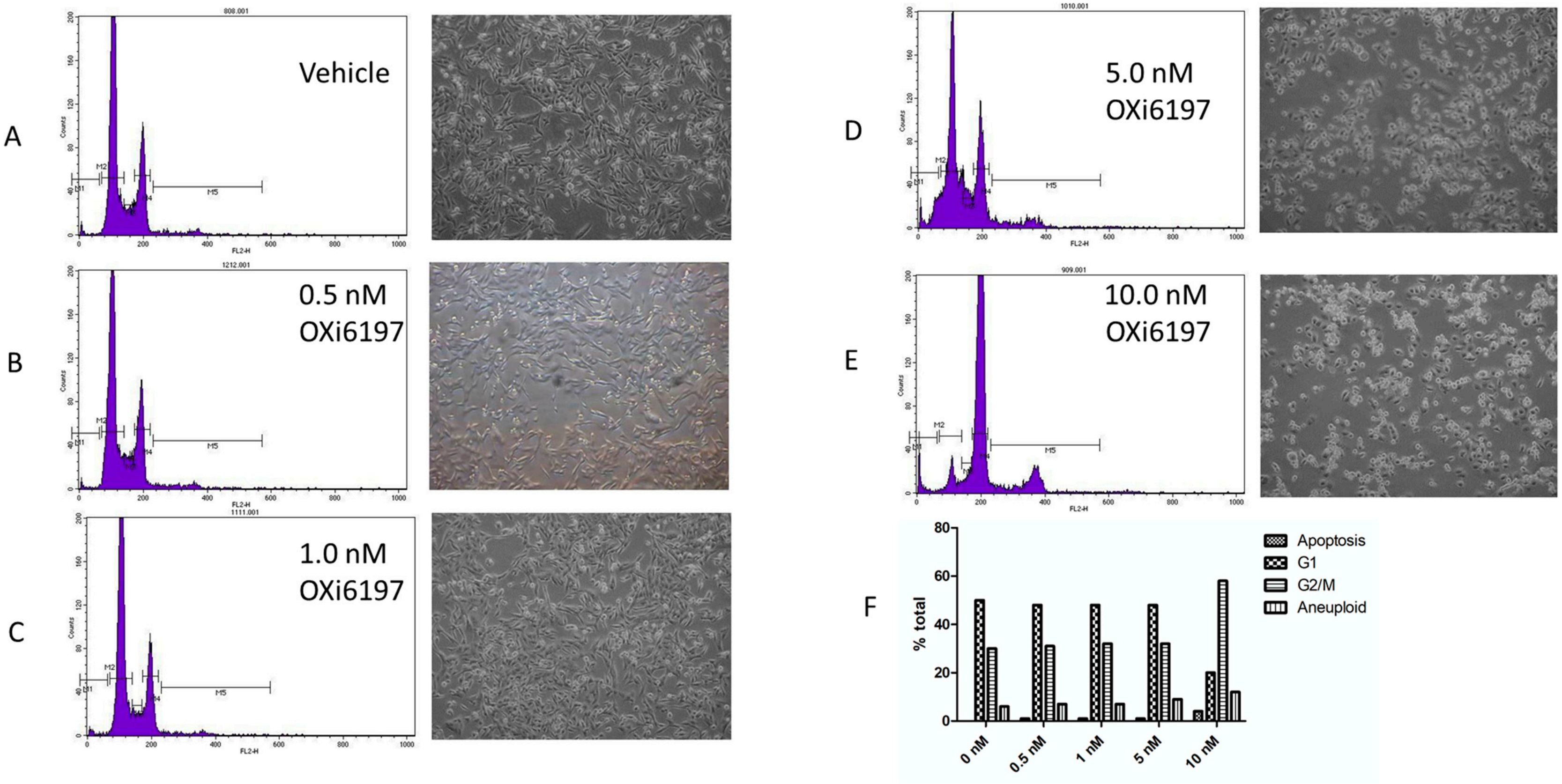

Figure S2. Flow cytometry of MDA-MB-231 cells treated with A) vehicle control, B) 0.5 nM, C) 1.0 nM, D) 5.0 nM, and E) 10.0 nM OXi6197. At 5 nM OXi6197 treatment (D) the cells were rounding, and at 10 nM (E) G2/M blockade was observed, as summarized in (F).

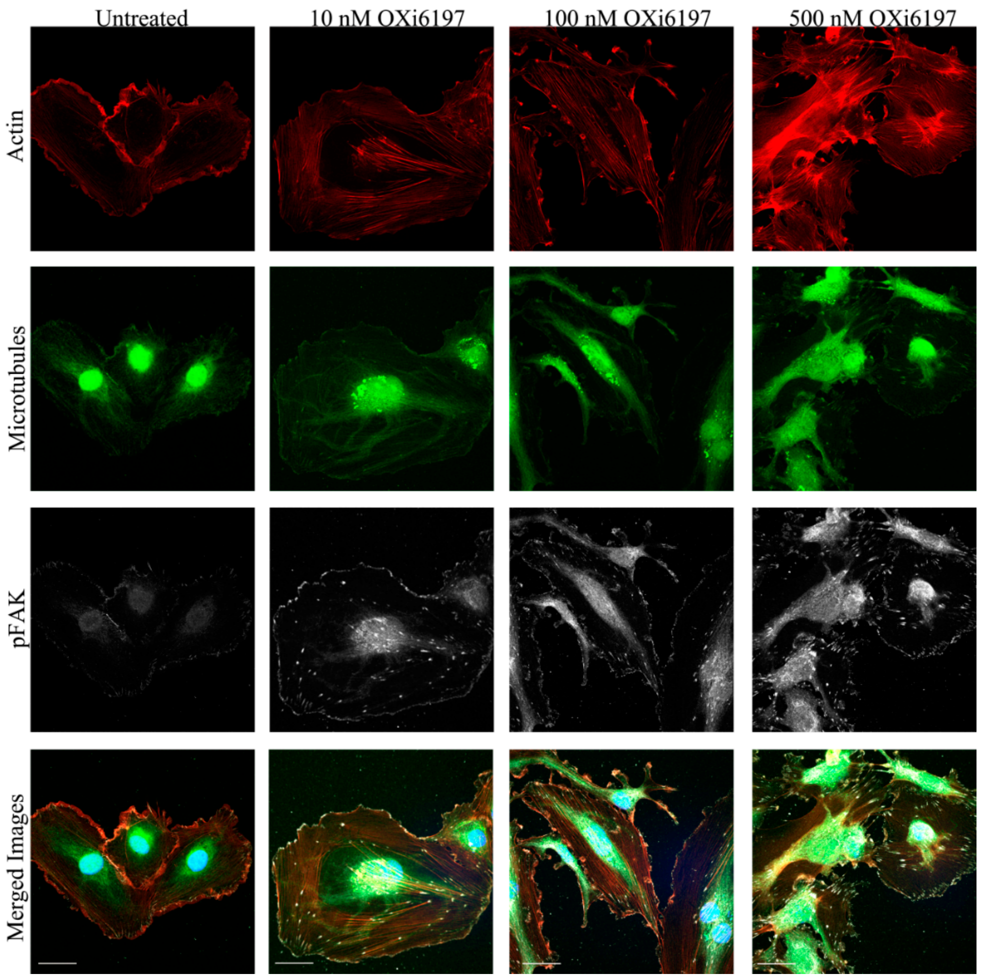

Figure S3: Effects of OXi6197 treatment on activated HUVECs: confocal microscopy. Representative confocal images demonstrating the concentration-dependent morphological effects of OXi6197 treatment on activated HUVECs. Monolayers of HUVECs growing on gelatin-coated coverslips were treated with vehicle, or 10 nM, 100 nM or 500 nM OXi6197 for 2 h. HUVECs were fixed and stained with Alexa Fluor™ 594-conjugated phalloidin (red, actin), anti- -tubulin antibody (green, microtubules), and anti-pFAKY397(Cy5 white, pFAK). The concentration dependent increase in focal adhesion kinase phosphorylation demonstrates focal adhesion formation.

Vehicle Control

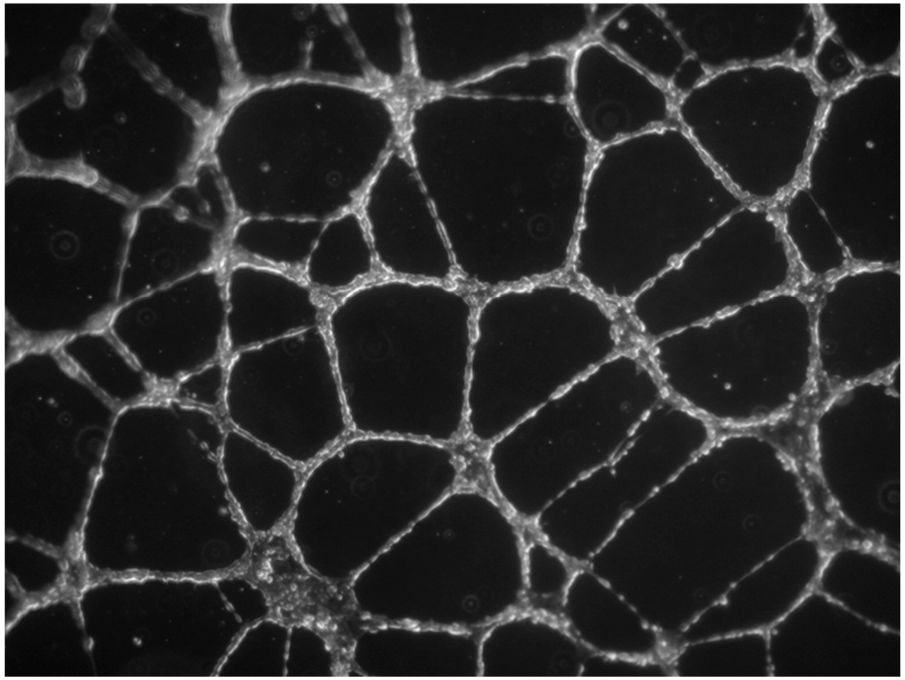

Figure S4: HUVEC capillary-like network established on Matrigel®

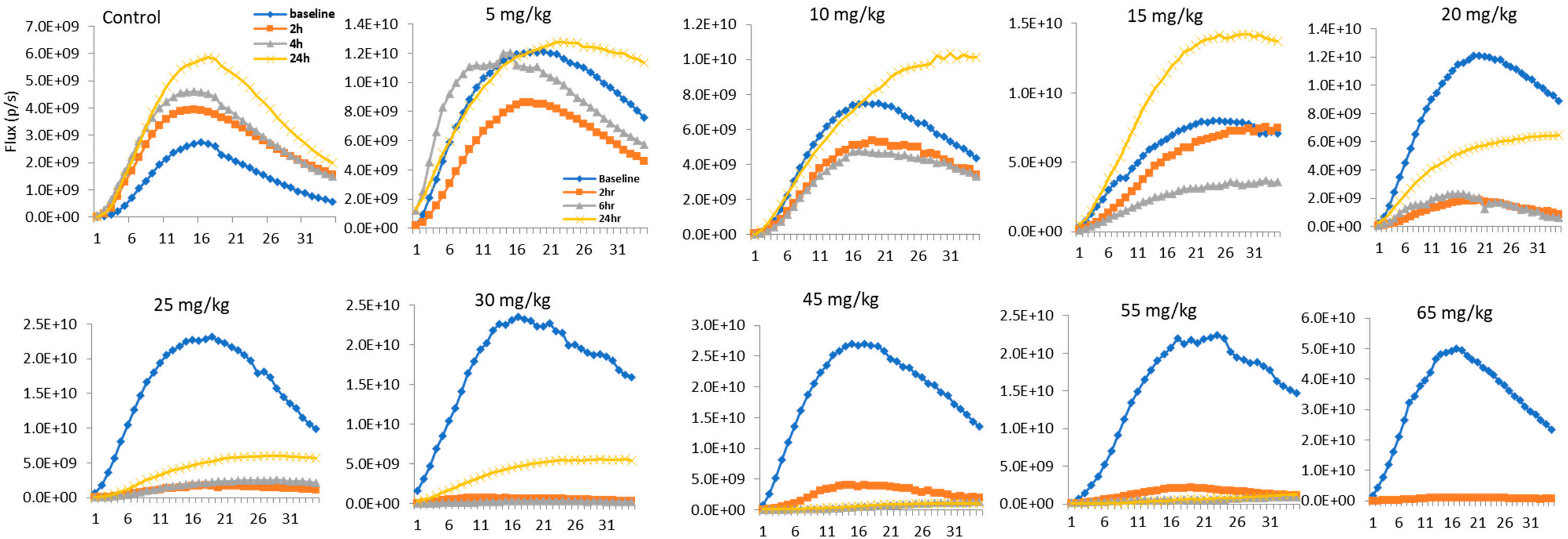

Figure S5. BLI observations of the MDA-MB-231-luc tumor dose response to OXi6197. BLI intensity curves for respective individual mice showed differential variations over a period of 35 mins following the administration of luciferin at baseline (blue), 2 h (orange), 4 or 6 h (grey) and 24 h (yellow) following each of the doses of OXi6197: respectively, saline control, 5 mg/kg, 10 mg/kg, 15 mg/kg, 20 mg/kg, 25 mg/kg, 30 mg/kg, 45 mg/kg 55 mg/kg and 65 mg/kg. Sequential time courses for the saline control indicated increased signal consistent with rapid tumor growth. Meanwhile, increasing doses of OXi6197 led to lower signal, as summarized in Supplementary Figure S6.

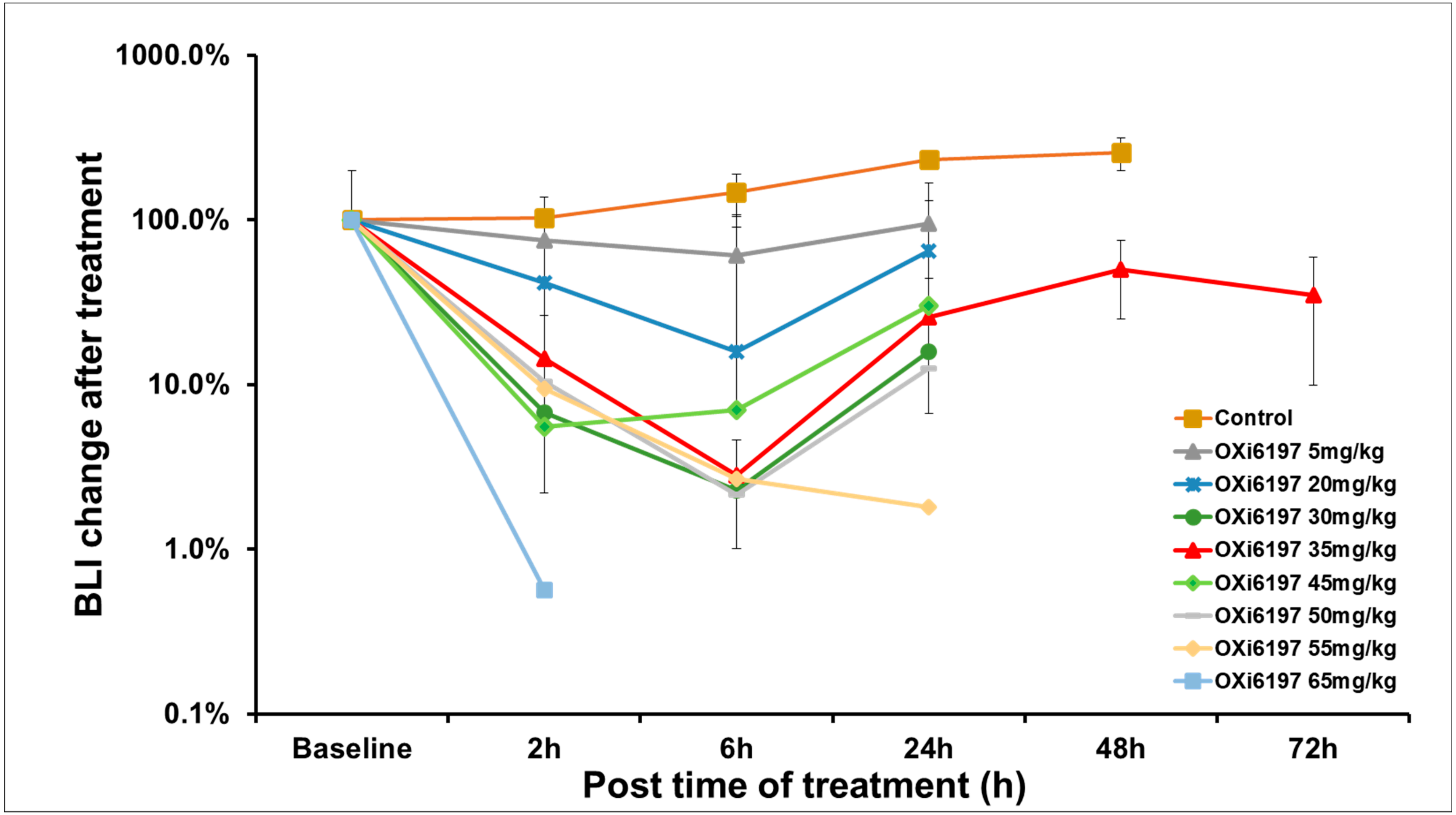

Figure S6: Comparison of relative vascular shutdown following increasing doses of OXi6197. Relative signals indicated differential dose responses following the administration of different doses of OXi6197 to MDA-MB-231-luc tumor-bearing NOD SCID mice: saline control (n = 5, brown square), 5 mg/kg (n = 2, grey triangle), 20 mg/kg (n = 4, blue cross), 30 mg/kg (n = 4, green circle), 35 mg/kg (n = 14, red triangle) and 45 mg/kg (n=3, green diamond), 50 mg/kg (n = 2, grey square), 55 mg/kg (n = 1, yellow triangle), and 65 mg/kg (n = 1, blue square). Individual data points are the mean ± SD of maximum intensity observed on the light emission curve.

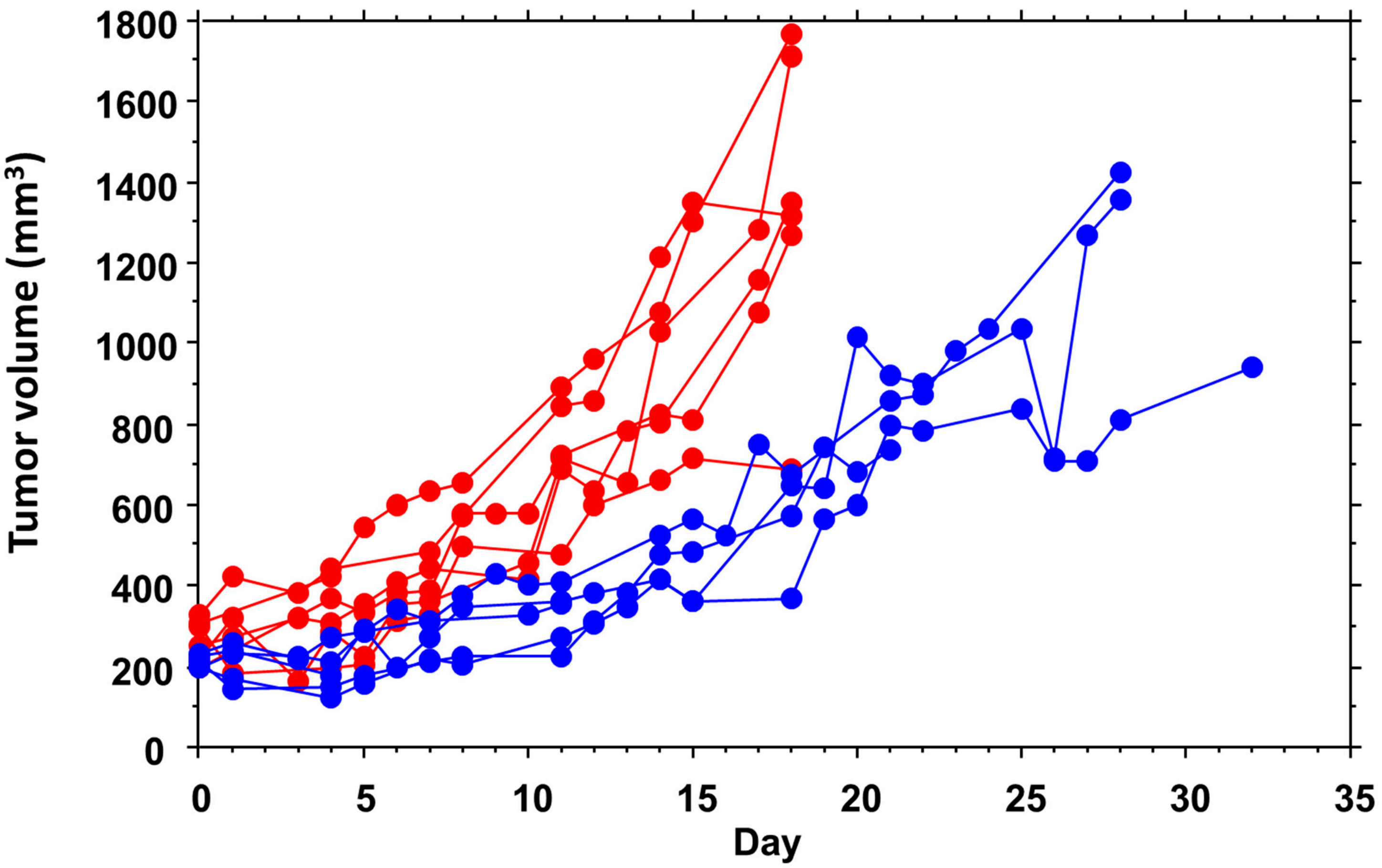

Figure S7: Growth curves for individual orthotopic MDA-MB-231-luc tumors receiving saline (red, controls; n=6) or OXi6197 (blue, 20 mg/kg twice weekly ; n=5). Individual tumors in each group showed highly consistent growth.

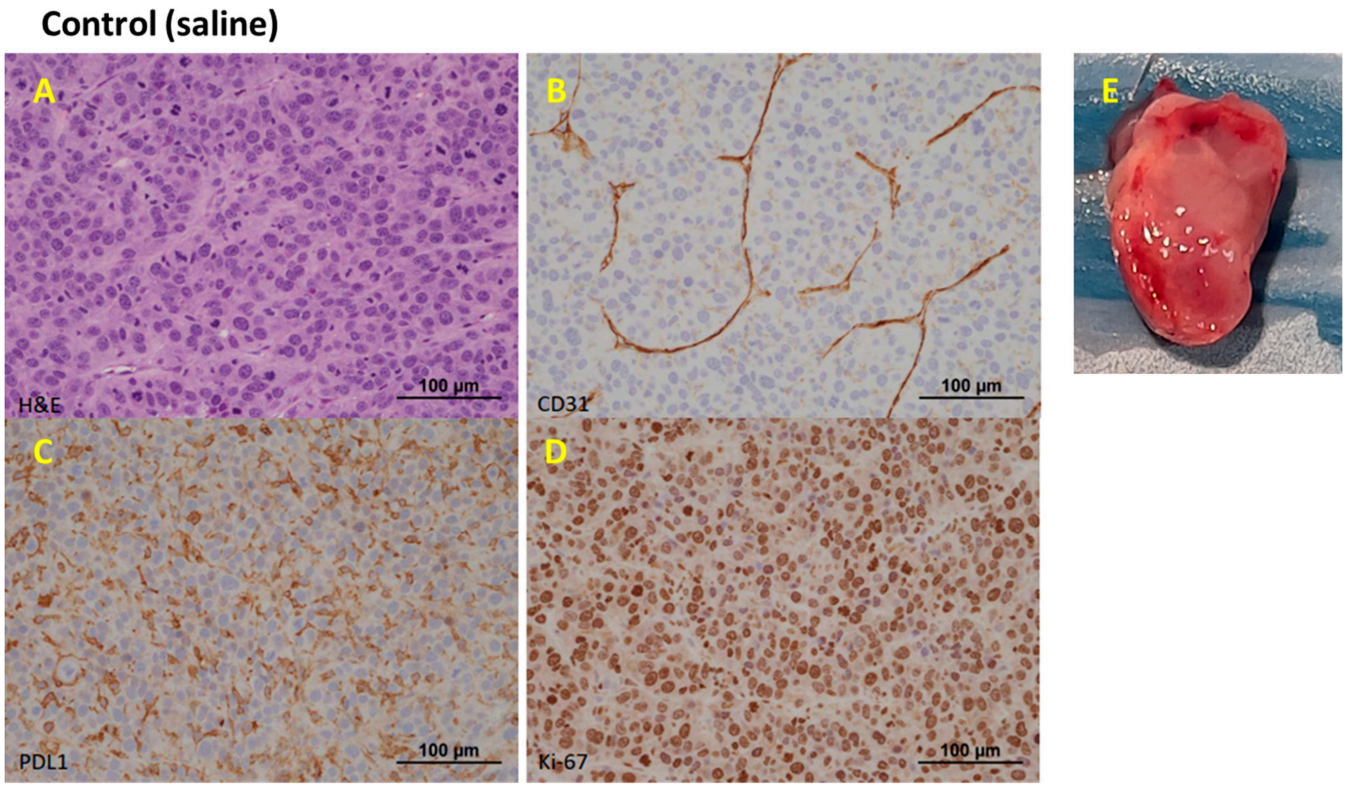

Figure S8: Representative histology images of control RENCA-luc tumor from selected areas of whole mount tumor. A) H&E showing viable pleomorphic tumor cells, B) CD31 IHC highlighting the thin vascular network, C) Tumor cells with prominent membranous and cytoplasmic PD-L1 expression, and D) Ki67 showing high proliferative activity, and E) Photograph of tissue slice at time of sacrifice and resection.

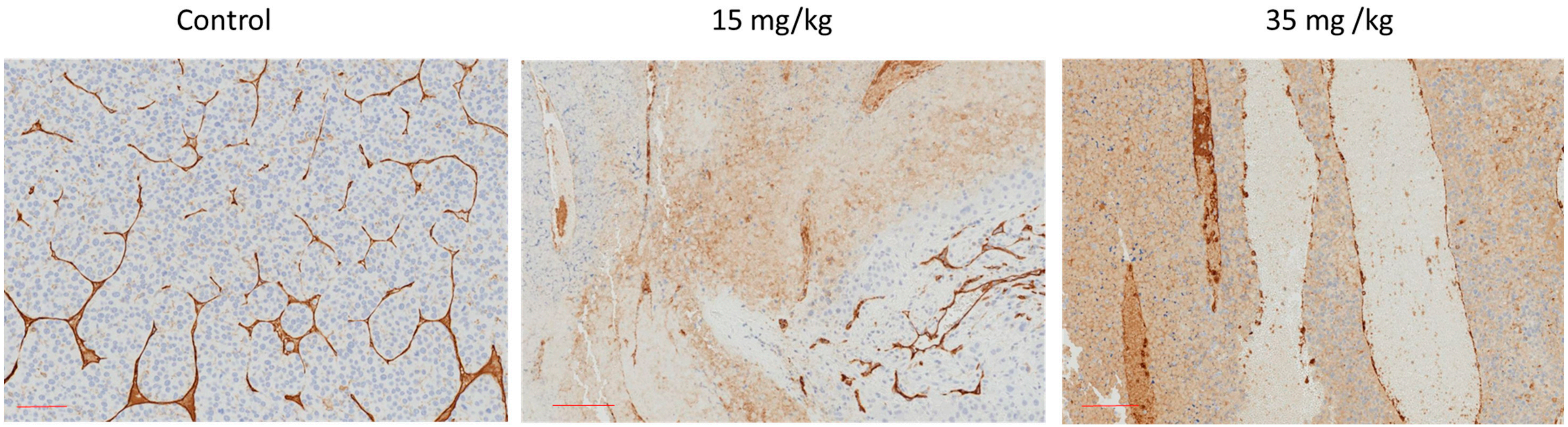

Figure S9: Representative histology images of CD31 IHC from RENCA-luc tumors A) highlighting the thin vascular network of control untreated tumors, B) Swollen vessels and pockets of remaining vasculature after 15 mg/kg OXi6197, and C) Massively swollen vessels after 35 mg/kg OXi6197. Tissues resected 24 h after administration of OXi6197, as for Figure 8. Red bar 100 μm.

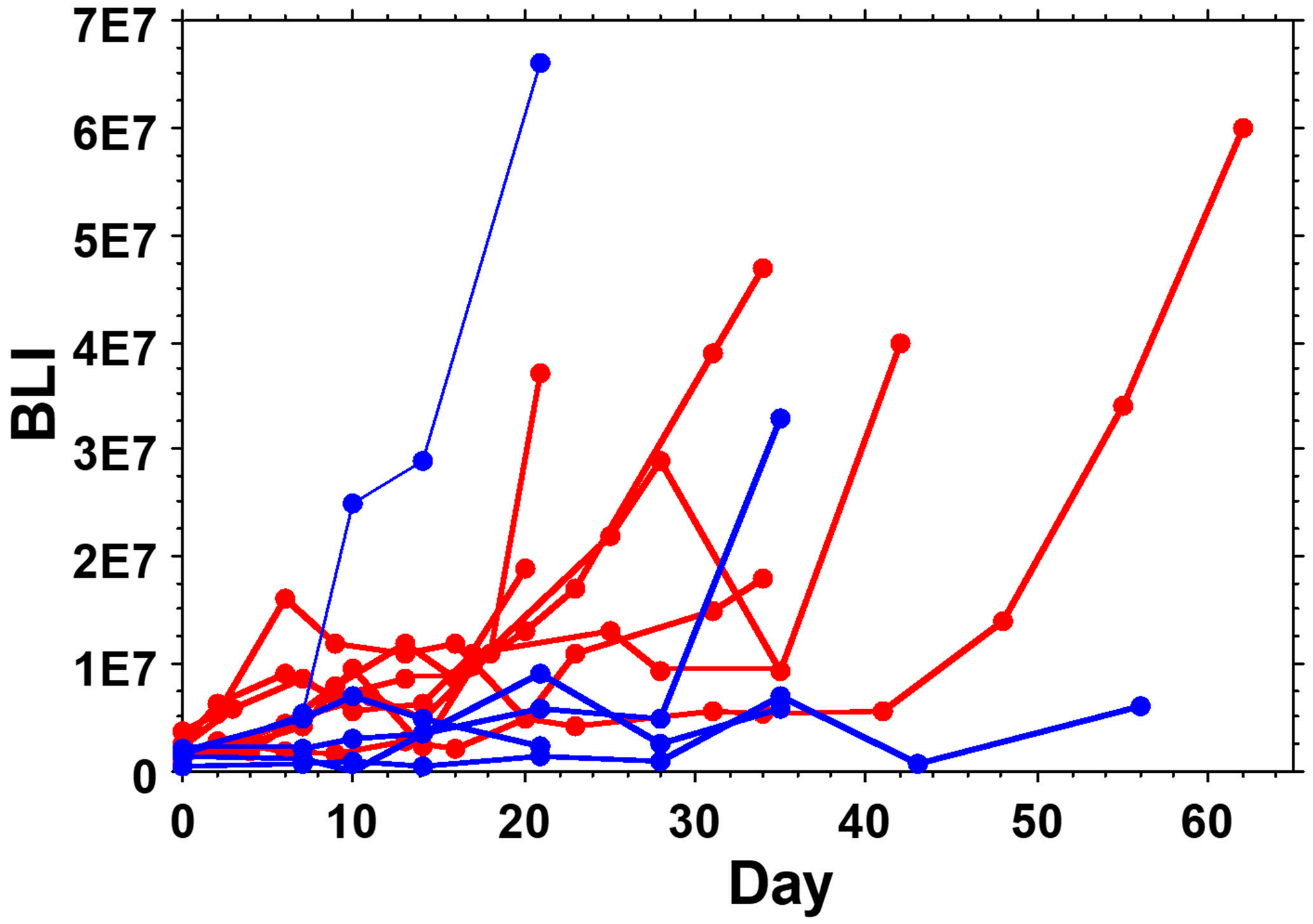

Figure S10: Growth curves based on BLI signal for individual orthotopic RENCA-luc tumors receiving saline (red, controls, n=7) or OXi6197 (blue, 35 mg/kg twice weekly, n=5). Treated mice generally showed slower growth and the obvious outlier is marked with asterisk.

© 2022 by the authors. Licensee MDPI, Basel, Switzerland. This article is an open access article distributed under the terms and conditions of the Creative Commons Attribution (CC BY) license (http://creativecommons.org/licenses/by/4.0/).
